# Pomegranate Peel Extract Alleviates Psoriasis-like Skin Lesions in Mice Through the Suppression of AhR-Activating Th17/IL-17 Axis and Neuronal-Related Pathways

**DOI:** 10.3390/ijms27125171

**Published:** 2026-06-07

**Authors:** Jiale Qi, Yujia Fu, Bing Ge, Xingkang Wu

**Affiliations:** Modern Research Center for Traditional Chinese Medicine, Key Laboratory of Chemical Biology and Molecular Engineering of Ministry of Education, Shanxi University, Taiyuan 030006, China; 18142419212@163.com (J.Q.); fuyujia134@163.com (Y.F.); gebing20010819@163.com (B.G.)

**Keywords:** pomegranate peel, psoriasis, AhR, neuronal-related pathways, Th17/IL-17 axis

## Abstract

Psoriasis is a refractory skin disease that is often accompanied by systemic comorbidities. While the efficacy of biologics in treating psoriasis is well-established, these treatments are also associated with frequent adverse reactions. Consequently, there is an urgent need to develop new treatment strategies for psoriasis. Pomegranate peel, which is widely used in skincare and protection, has not been fully studied for its therapeutic potential in psoriasis. This article investigates the therapeutic effectiveness of pomegranate peel ethanol extract (PEE) on psoriasis. A total of 2872 compounds were identified in PEE. The topical application of PEE alleviated primary psoriasis-like skin lesions and reduced lesion recurrence in mice. PEE simultaneously activates AhR and inhibits the Th17/IL-17 axis and neuronal-related pathways, which may serve as a mechanism for PEE in the treatment of psoriasis. In conclusion, our study establishes that PEE may serve as a novel therapeutic agent for psoriasis, with a unique mechanism of action that simultaneously activates AhR and suppresses neuronal-related pathways.

## 1. Introduction

Psoriasis is a chronic, refractory, and recurrent skin disease that affects 1–3% of the global population [1]. Its primary pathological manifestations include incomplete epidermal keratinization, dermal vascular proliferation, and infiltration of inflammatory cells [1]. Although the pathological mechanisms underlying psoriasis have not been fully elucidated, dysregulation of the IL-23/Th17 axis is recognized as a core pathological mechanism [1]. In psoriatic lesions, dendritic cells and macrophages produce IL-23, which activates Th17 cells and drives the release of inflammatory cytokines, including IL-17A, IL-17F, IL-22, IL-6, and TNF-α. IL-17A, IL-17F, and IL-22 act on keratinocytes, leading to pathological changes associated with psoriasis, including epidermal hyperplasia and hyperkeratosis [2]. Within the skin’s inflammatory microenvironment, keratinocytes can produce IL-23 and other inflammatory factors and chemokines, thereby forming an IL-23/Th17 positive feedback loop that amplifies and exacerbates the chronic inflammatory process of psoriasis [2]. Furthermore, recent studies indicate that the nervous system regulates immune responses and keratinocyte proliferation in psoriasis. Cutaneous sensory innervation and neuro-immune crosstalk play important roles in the pathogenesis of psoriasis [3]. Sensory nerve endings release neuropeptides, including substance P and CGRP, which interact with keratinocytes and immune cells to promote neurogenic inflammation and form a positive feedback loop with the IL-23/Th17/IL-17 axis [3]. In addition, inflammatory cytokines such as IL-31 activate sensory neurons, further aggravating pruritus, epidermal hyperproliferation, and inflammatory infiltration [4]. Thus, sensory nerves serve as key regulators in psoriasis inflammation and lesion progression, and targeting neuro-immune crosstalk represents a promising therapeutic strategy.

In addition to presenting as scaly erythema, psoriasis is often closely associated with systemic comorbidities such as psoriatic arthritis, non-alcoholic fatty liver disease, cardiovascular diseases, and chronic kidney disease [5]. Approximately 57.9% of psoriasis patients have at least one comorbidity [5]. Psoriasis comorbidities encompass multiple systems, including the cardiovascular, endocrine, metabolic, immune, and nervous systems [5]. These comorbidities not only affect the progression and severity of psoriasis but also influence treatment options and outcomes for patients [5]. The occurrence and severity of psoriasis comorbidities are clearly correlated with the severity of psoriasis-like skin lesions [5]. Developing drugs that can not only treat psoriatic lesions but also prevent the occurrence of comorbidities has become a new trend in psoriasis drug research.

The treatment strategy for psoriasis depends primarily on disease severity (mild or moderate-to-severe), comorbidities, and other medical conditions. Mild-to-moderate cases are mainly treated with topical therapies (e.g., glucocorticoids, vitamin D analogues, and phototherapy), while moderate-to-severe cases or those with psoriatic arthritis require systemic treatments. Traditional small-molecule drugs include methotrexate, cyclosporine A, and retinoids; however, these drugs have significant toxic side effects [6,7]. Novel small molecules such as dimethyl fumarate and apremilast exhibit immunomodulatory effects but may cause gastrointestinal adverse effects [6,7]. Significant breakthroughs in biologics over the past few years have provided more effective options for psoriasis treatment, including inhibitors targeting TNF-α, IL-23, and IL-17, as well as other pathways [8,9]. Despite the substantial advances in biologic therapies for psoriasis, their potential side effects and infection risks have prompted the search for safer, more effective alternative therapies. Therefore, the development of therapeutic drugs for psoriasis remains an unmet clinical need.

The pomegranate peel accounts for as much as 50% of the entire fruit and is considered a by-product generated during fruit processing [10]. Developing and utilizing pomegranate peel can enhance the economic benefits of the pomegranate industry and optimize the use of pomegranate resources [10]. The relationship between its traditional medicinal value and modern pharmacological research is currently attracting widespread attention. Pomegranate peel has long been used in traditional medicine to address a variety of health issues, ranging from digestive disorders such as diarrhea to parasitic infections [11]. In recent years, pomegranate peel has been extensively explored as a natural alternative to synthetic preservatives in meat products due to its notable antibacterial, anti-inflammatory, and antioxidant properties [12]. Additionally, pomegranate peel, rich in antioxidants, combats oxidative stress by scavenging free radicals, making it a potential approach to reduce the incidence of chronic diseases and delay aging [13]. Anti-inflammatory compounds in pomegranate peel have also demonstrated efficacy in alleviating inflammation-related diseases [14]. In summary, pomegranate peel not only serves as an effective natural preservative but also shows broad potential for application in food, medicine, and cosmetics due to its antibacterial, anti-inflammatory, and antioxidant properties.

More excitingly, pomegranate peel is widely used to treat inflammatory skin diseases [15]. The compounds abundant in pomegranate peel extract have shown promise in promoting skin wound healing [16]. Pomegranate peel extract exerts targeted therapeutic effects against psoriasis through well-defined molecular mechanisms: suppression of inflammatory cascades, regulation of keratinocyte hyperproliferation, antimicrobial activity against pathogens, and mitigation of oxidative stress [17]. Recent studies have demonstrated the significant efficacy of punicalagin (PUN), a key bioactive compound in pomegranate peel extract, in alleviating psoriatic symptoms [18]. However, the effects of pomegranate peel on psoriasis remain incompletely understood.

This study investigated the therapeutic effectiveness of pomegranate PEE on psoriasis. Firstly, plant metabolomics was employed to analyze the chemical composition of PEE. The therapeutic effects of PEE on psoriasis were then evaluated using primary and recurrent mouse models of the condition. Subsequently, transcriptomics, IPA, and network pharmacology were used to elucidate the mechanism of action of PEE in the treatment of psoriasis. These findings provide new insights into the therapeutic effectiveness of pomegranate peel for psoriasis, which is of great significance for the development of the pomegranate industry.

## 2. Results

### 2.1. Uncovering the Chemical Composition of PEE

**Figure 1:**
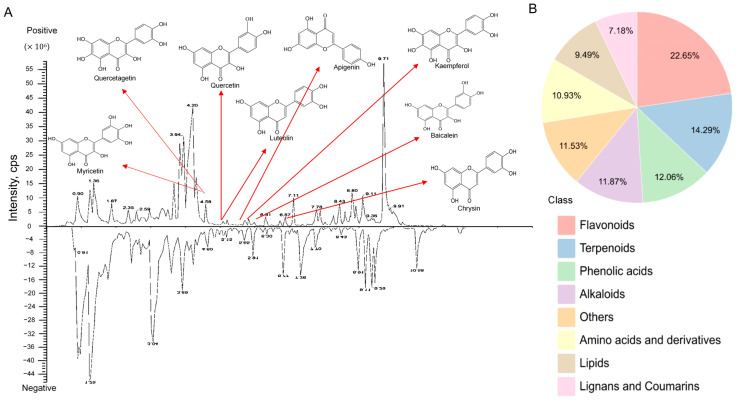
Chemical composition analysis of PEE. (**A**) TIC of the PEE. The x-axis represents the retention time of metabolite detection, while the y-axis indicates the ion current intensity (unit: cps, counts per second) of ion detection. The peaks of eight flavonoids are labeled. (**B**) The structural types of compounds in PEE.

To quantitatively identify the chemical components of PEE, the metabolomics analysis was applied. UPLC-MS/MS analysis of PEE was performed in both positive and negative ion modes. Based on fragment matching with database records, 2872 compounds were identified, and their relative abundances were determined (Table S3). The total ion chromatogram (TIC) of the identified compounds is shown in Figure 1A. Based on the structural types of the compounds, these compounds were classified into flavonoids, terpenoids, phenolic acids, alkaloids, amino acids, lipids, lignans, coumarins, and others, accounting for 22.65%, 14.29%, 12.06%, 11.87%, 11.53%, 10.93%, 9.49%, and 7.18% of the PEE, respectively (Figure 1B). In the PEE, the content of flavonoids was the highest (Figure 1B). Additionally, the peaks of eight flavonoids, including myricetin, quercetagetin, quercetin, luteolin, apigenin, kaempferol, baicalein, and chrysin, which were investigated in the latter part of the article, are labeled in the TIC diagram (Figure 1A).

### 2.2. PEE Alleviated Psoriasis-like Lesions in Mice

**Figure 2:**
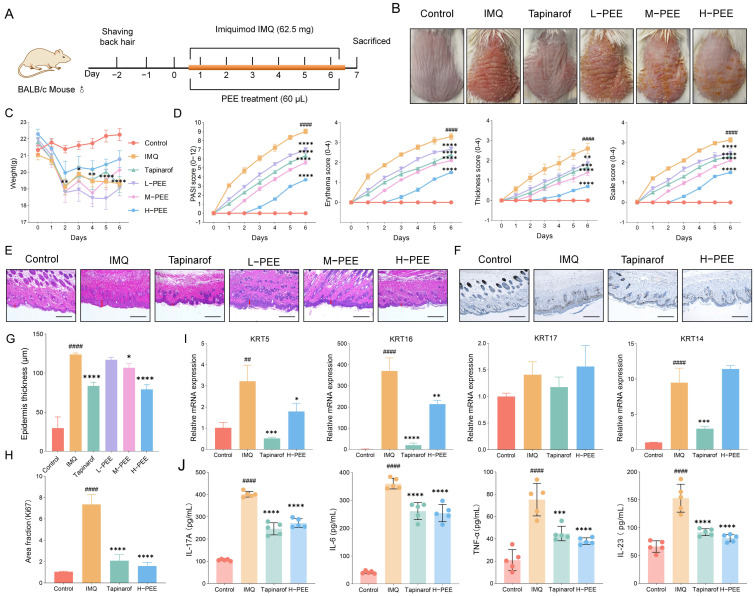
PEE alleviated psoriasis-like skin lesions in mice. (**A**) The workflow for the construction and treatment of psoriasis mouse models. (**B**) Representative images of psoriasis-like skin lesions in mice. (**C**) Changes in mouse body weight over the experimental period. (**D**) Quantification of the PASI total score, erythema, scaling, and skin thickness of psoriasis-like skin lesions. (**E**) H&E staining images of psoriasis-like skin lesions in mice. Red lines represent the thickness of the epidermis. Scale bar: 300 μm. (**F**) The expression of Ki67 in psoriasis-like skin lesions in mice. The skin samples were subjected to IHC analysis. Scale bar: 300 μm. (**G**) Quantification of epidermal thickness based on H&E staining images, with epidermal thickness indicated by lines in the H&E staining images. (**H**) Quantification of Ki67 expression based on IHC images. (**I**) The mRNA expression levels of KRT5, KRT16, KRT17, and KRT14 in psoriasis-like skin lesions. (**J**) ELISA assay for IL-17A, IL-6, TNF-α, and IL-23 expression levels in psoriasis-like skin lesions. n = 5 samples per group; data are expressed as mean ± SD. ## *p* < 0.01, #### *p* < 0.0001, compared to control, * *p* < 0.05, ** *p* < 0.01, *** *p* < 0.001, **** *p* < 0.0001, compared to IMQ. Statistical significance was calculated using two-way ANOVA (**C**,**D**) and one-way ANOVA (**G**–**J**).

To investigate the therapeutic efficacy of PEE in psoriasis, mice with IMQ-induced psoriasis-like skin lesions were utilized (Figure 2A). Compared with the control group, the mice in the IMQ group exhibited decreased body weight and increased scores for PASI, erythema, thickness, and scaling of psoriatic lesions (Figure 2B–D). These findings confirm the successful establishment of the psoriasis-like model. Consistent with tapinarof, PEE increased the body weight of psoriatic mice in a concentration-dependent manner and reduced all PASI subscores (erythema, scaling, epidermal thickness) as well as the total score for psoriatic lesions (Figure 2B–D). Subsequently, HE staining showed that PEE reduced epidermal thickness in psoriasis-like skin lesions of mice in a concentration-dependent manner (Figure 2E,F). Among the three PEE doses, the high-dose PEE (H-PEE) showed the best therapeutic effect, so H-PEE was used for further studies on its anti-psoriasis effect.

Furthermore, Masson staining was performed to investigate dermal changes in psoriatic lesions. The collagen fibers in the dermis were reduced in the IMQ group, and increased after H-PEE treatment (Figure S1), indicating that PEE remodeled the dermal structure of psoriatic lesions. Ki67 is a marker of keratinocyte proliferation, and the results of the IHC analysis showed that H-PEE significantly reduced the Ki67 expression, revealing that H-PEE inhibited the keratinocyte proliferation in psoriatic lesions (Figure 2G,H). In addition, the expression of psoriasis-related markers (Ki67, KRT5, KRT16, KRT17, and KRT14) was detected. The results showed that H-PEE could inhibit the expression of KRT5 and KRT16, thereby improving the abnormal proliferation of keratinocytes in psoriatic lesions (Figure 2I). To further investigate the regulatory role of H-PEE in psoriasis-related inflammatory responses, we measured the levels of key inflammatory cytokines (IL-17A, IL-6, TNF-α, and IL-23) in lesion tissues via ELISA. The results showed that compared with the model group, H-PEE treatment significantly reduced the expression of these cytokines (Figure 2J), further confirming its therapeutic efficacy at the molecular level. In summary, the PEE ameliorated psoriatic lesions in mice, as evidenced by histopathological and molecular marker analyses.

### 2.3. H-PEE Alleviated the Relapse of Psoriatic Lesions in Mice

**Figure 3:**
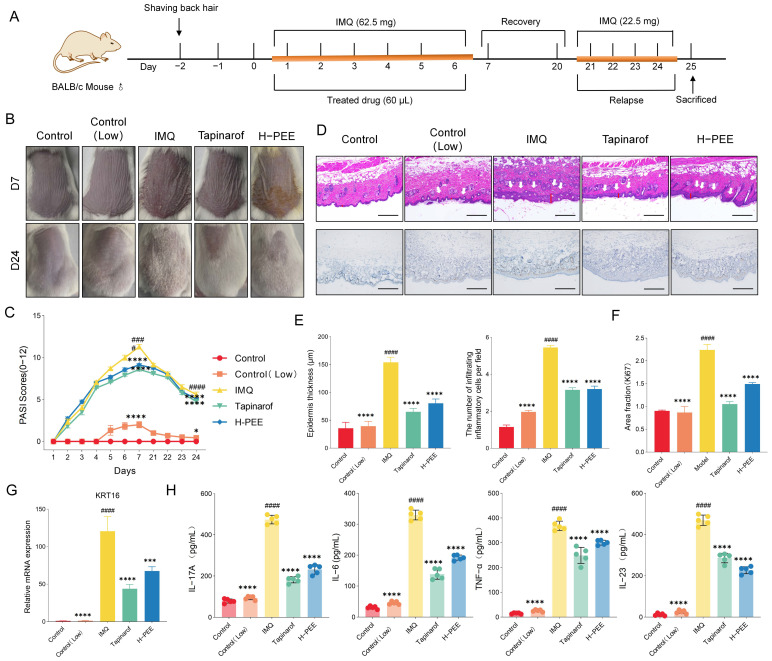
H-PEE alleviated the recurrence of psoriasis-like skin lesions in mice. (**A**) The workflow for constructing a recurrent psoriasis model in mice. (**B**) Representative images of the recurrent psoriasis-like skin lesions in mice. (**C**) Quantification of the PASI total score of psoriasis-like skin lesions over the experimental period. (**D**) H&E staining images and Ki67 IHC analysis images of recurrent psoriasis-like skin lesions in mice. Epidermal thickness is indicated by red lines in the H&E-stained images, and infiltrating immune cells are marked with white arrows. Scale bar: 300 μm. (**E**) Quantification of epidermal thickness and inflammatory cell infiltration based on H&E staining images. (**F**) Quantification of Ki67 expression based on IHC images. (**G**) The mRNA expression levels of KRT16 in psoriasis-like skin lesions. (**H**) ELISA assay for IL-17A, IL-6, TNF-α, and IL-23 expression levels in psoriasis-like skin lesions. Scale bar = 300 μm, n = 5 samples per group; data are expressed as mean ± SD. # *p* < 0.05, ### *p* < 0.001, #### *p* < 0.0001 compared to control; * *p* < 0.05, *** *p* < 0.001, **** *p* < 0.0001 compared to IMQ. Statistical significance was calculated using Two-way ANOVA (**C**) and One-way ANOVA (**E**–**H**).

Given that disease recurrence is a challenging issue in psoriasis treatment, the relapse of psoriatic lesions treated with H-PEE was investigated. After a 7-day treatment period and a 14-day recovery period, a recurrent psoriasis model was established in mice using a lower IMQ dose (Figure 3A). The model group, which received low-dose IMQ to induce recurrence of psoriatic lesions, exhibited weight loss and psoriatic lesions (erythema and scaling), whereas the control group did not (Figure 3B–D). In the tapinarof and H-PEE groups, weight loss and psoriatic skin erythema/scaling were less pronounced (Figure 3B–D). H&E staining revealed that H-PEE treatment significantly reduced epidermal thickness and immune cell infiltration compared to the low IMQ-induced recurrence group (Figure 3B–D). Ki67 immunohistochemistry showed a marked decrease in Ki67-positive keratinocytes in H-PEE-treated mice (Figure 3E,F). To further confirm the molecular-level inhibition of psoriasis recurrence by H-PEE, we examined the expression of psoriasis-related marker molecules KRT16, IL-17A, IL-6, TNF-α, and IL-23. The qPCR results demonstrated that H-PEE effectively downregulated the abnormally elevated expression of KRT16 in the recurrent psoriatic lesions (Figure 3G). Meanwhile, ELISA analysis revealed that H-PEE treatment significantly reduced the concentrations of pro-inflammatory factors IL-17A, IL-6, TNF-α, and IL-23 in these lesions (Figure 3H). These results indicate that H-PEE treatment for psoriasis can effectively prevent recurrence, demonstrating that H-PEE not only ameliorates active psoriasis-like skin lesions but also prevents their recurrence, highlighting its significant value as a long-term treatment strategy for psoriasis.

### 2.4. PEE Regulated the Gene Expression Profiles of Psoriatic Lesions

**Figure 4:**
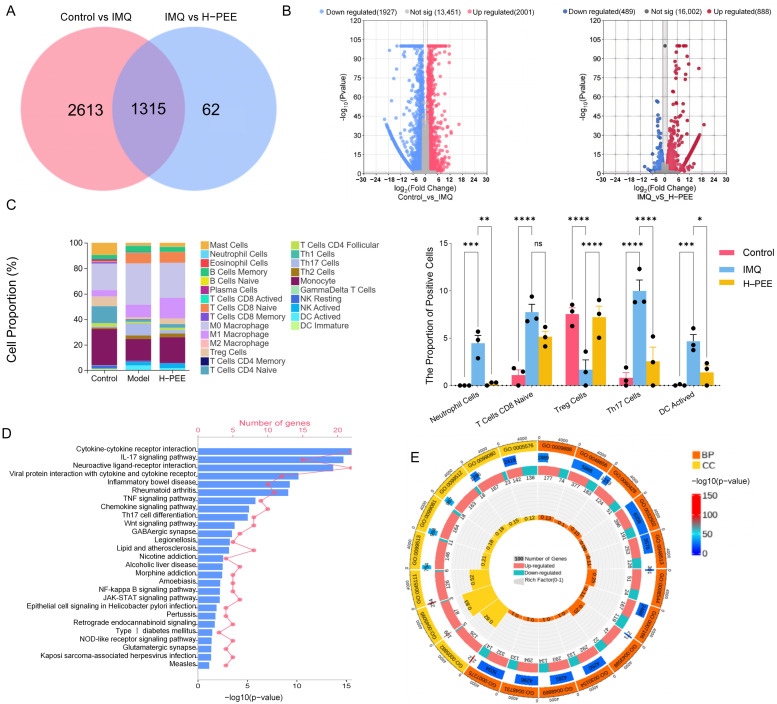
Transcriptome analysis of psoriasis-like skin lesions in mice. (**A**) Venn diagram of differentially expressed genes (DEGs). (**B**) Volcano plots of DEGs. (**C**) Analysis of immune cell infiltration in psoriatic lesions using ImmuCellAI-mouse tool. (**D**) GO term enrichment analysis of DEGs. (**E**) Kyoto Encyclopedia of Genes and Genomes (KEGG) pathway enrichment analysis of DEGs. * *p* < 0.05, ** *p* < 0.01, *** *p* < 0.001, **** *p* < 0.0001, ns: no significant difference (*p* > 0.05) vs. IMQ group.

To further elucidate the potential therapeutic mechanisms of H-PEE in psoriasis, transcriptomic analysis was performed to investigate gene expression profiles in psoriatic lesions treated with H-PEE. Compared to the control group, the IMQ group exhibited 3928 differentially expressed genes (DEGs), including 2001 upregulated genes and 1927 downregulated genes (Figure 4A,B and Table S4). In contrast, the H-PEE group showed 1377 DEGs, including 888 upregulated and 489 downregulated genes, compared to the IMQ group (Figure 4A,B). First, the ImmuCellAI-mouse tool was used to analyze gene expression profiles and investigate the regulatory effects of H-PEE on the immune microenvironment in psoriatic lesions. Compared with the control group, immune cells in the skin lesions of IMQ-treated mice exhibited a typical pro-inflammatory state, characterized by significantly increased infiltration of neutrophils, activated dendritic cells, and Th17 cells, while Treg cells, which have immunosuppressive functions, were markedly reduced (Figure 4C). KEGG enrichment analysis indicated that the DEGs in the H-PEE group were primarily enriched in autoimmune-related signaling pathways, including cytokine-cytokine receptor interactions, the IL-17 signaling pathway, the TNF signaling pathway, Th17 cell differentiation, the NF-kB signaling pathway, and the JAK-STAT signaling pathway, all of which are involved in the pathological process of psoriasis (Figure 4D). Further GO enrichment analysis revealed that, compared to the IMQ group, the differentially expressed genes (DEGs) in the H-PEE group were primarily enriched in keratin filaments, intermediate filaments, intermediate filament cytoskeleton, epidermis development, and skin development (Figure 4E and Table S5). Therefore, the transcriptomic results indicated that PEE modulated the infiltration of pro-inflammatory immune cells in psoriatic lesions and the signaling pathways involved in psoriasis.

### 2.5. PEE Reversed Psoriasis-Related Canonical Pathways, Biological Functions, and Upstream Regulators

**Figure 5:**
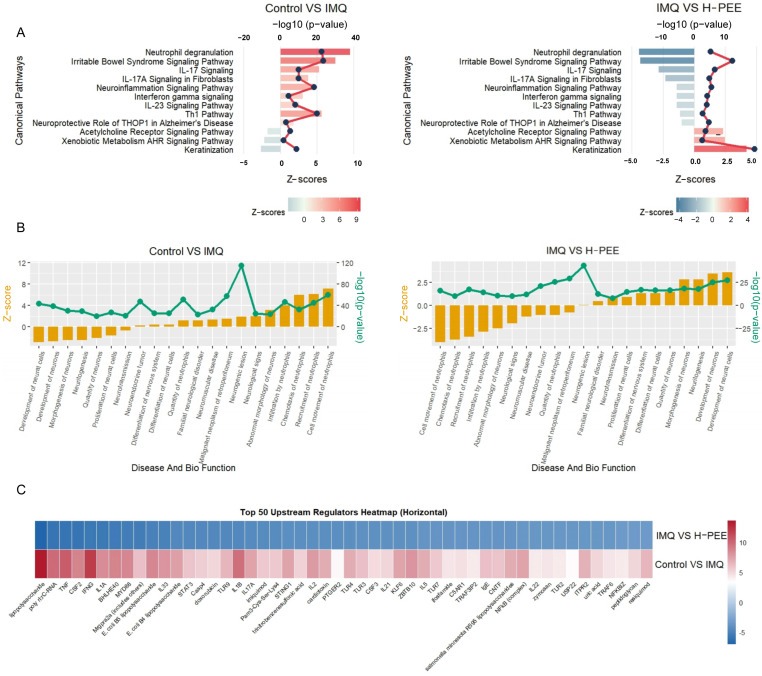
IPA illustrating the perturbation effects of H-PEE on psoriatic pathways, biological functions, and upstream regulators. (**A**) The regulatory effects of H-PEE on canonical pathways. (**B**) The regulatory effects of H-PEE on biological functions. (**C**) The regulatory effects of H-PEE on upstream regulators.

Furthermore, IPA software (v4.13.1) was used to analyze the regulatory activity of H-PEE on psoriasis-related canonical pathways, biological functions, and upstream regulators. In IPA, the z-score indicates the activation or inhibition of the pathways, biological functions, and upstream regulators. In IMQ-induced psoriatic lesions, nine pathways were activated, including neutrophil degranulation, irritable bowel syndrome signaling pathway, IL-17 signaling, IL-17A signaling in fibroblasts, neuroinflammation signaling pathway, interferon gamma signaling, IL-23 signaling pathway, Th1 pathway, and the neuroprotective role of THOP1 in Alzheimer’s disease. Additionally, three pathways were inhibited: the acetylcholine receptor signaling pathway, the AhR signaling pathway in xenobiotic metabolism, and keratinization (Figure 5A). However, H-PEE reversed the IMQ-induced alterations in these pathways. Interestingly, KEGG enrichment and IPA canonical pathway analysis revealed that the H-PEE exerted a strong regulatory effect on neuronal-related pathways, including neuroactive ligand–receptor interaction, neuroinflammation signaling pathway, the neuroprotective role of THOP1 in Alzheimer’s disease, and the acetylcholine receptor signaling pathway. These neuronal-related pathways are not the classic pathological mechanisms of psoriasis, which are primarily immune-related pathways. Therefore, we further analyzed the effects of the drug on neuronal-related pathways. In IMQ-induced psoriatic lesions, seven neuronal-related biofunctions were inhibited, while fourteen were activated; however, H-PEE reversed these altered biofunctions (Figure 5B). Meanwhile, the upstream regulatory factors causing changes in gene expression were analyzed. In IMQ-induced psoriatic lesions, approximately half of the top 50 activated upstream regulators were pathogenic factors of psoriasis. However, in PEE-treated psoriatic lesions, these upstream regulators were suppressed (Figure 5C). In summary, IPA elucidated the activated or inhibitory activities of PEE on psoriatic pathways, biofunctions, and upstream regulators for the treatment of psoriasis.

### 2.6. PEE Blocked the Th17/IL-17 Axis in Psoriasis-like Skin Lesions

**Figure 6:**
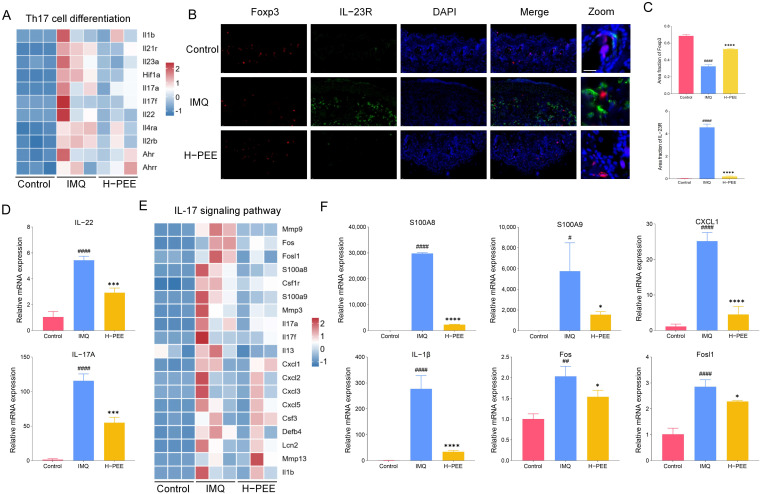
H-PPE blocked the Th17/IL-17 axis in psoriasis-like skin lesions. (**A**) Heatmap of gene expressions involved in the Th17 cell differentiation pathway. The expression levels were derived from transcriptomic data. (**B**) The Immunofluorescence assay of Th17 and Treg cells in skin tissue. Th17 and Treg cells were marked by IL-23R and Foxp3 foci, respectively. Scale bar: 20 μm. (**C**) Relative quantitative analysis of Th17 and Treg cells based on the immunofluorescence assay. (**D**) The mRNA expression levels of IL-17A and IL-22 in skin tissue. n = 5 samples per group; data are expressed as mean ± SD. (**E**) Heatmap of gene expression involved in the IL-17 signaling pathway. The expression levels were derived from transcriptomic data. (**F**) The mRNA expression levels of S100A8, S100A9, CXCL1, IL-1β, Fos, and Fosl1 in skin tissue. n = 5 samples per group; data are expressed as mean ± SD. # *p* < 0.05, ## *p* < 0.01, #### *p* < 0.0001, compared to control; * *p* < 0.05, *** *p* < 0.001, **** *p* < 0.0001, compared to IMQ. Statistical significance was calculated using one-way ANOVA (**C**,**D**,**F**).

The KEGG and CIBERSORT analyses of transcriptomics indicated that H-PEE significantly modulated the IL-17 signaling pathway and the Th17/Treg cell balance, which are core pathways of the IL-23–IL-17 immune axis. IL-23 activates Th17 cells by binding to IL-23R and promotes IL-17A secretion, thereby constituting the core pathogenic pathway of psoriasis. As a membrane surface protein, IL-23R exhibits more stable expression and more reliable detection in tissue sections than the intracellular factor IL-17. Therefore, IL-23R was chosen as the marker for Th17 cells in this study. The transcriptomic results showed that genes associated with the IL-17 signaling pathway were upregulated in the model group, whereas H-PEE downregulated their transcription levels (Figure 6A). Furthermore, the results of RT-qPCR validation of the transcriptomics indicated that H-PEE inhibited the upregulation of S100A8, S100A9, CXCL1, IL-1β, Fos, and Fosl1 expression induced by IMQ (Figure 6B). The transcriptomic and RT-qPCR results suggest that H-PEE inhibits the IL-17 signaling pathway in psoriasis-like skin lesions. Additionally, transcriptomic data highlighted enrichment for Th17 cell differentiation, an upstream pathway of the IL-17 signaling pathway. Transcriptomic results revealed that H-PEE decreased the expression of key genes involved in Th17 cell differentiation (Figure 6C). Subsequently, RT-qPCR experiments showed that H-PEE reduced the elevated expression of IL-17 and IL-22 induced by IMQ, both of which are expressed by Th17 cells (Figure 6D). Furthermore, Th17 cells and their opposing regulatory T cells (Treg) were detected using fluorescent immunohistochemistry. The results revealed a significant reduction in FOXP3+ Treg cells and an increase in IL-23R+ Th17 cells in the IMQ group compared to the control group (Figure 6E,F). Notably, H-PEE significantly increased Treg cells and decreased Th17 cells, indicating that H-PEE inhibited the differentiation of Th17 cells and promoted the differentiation of Treg cells (Figure 6E,F). Overall, these results indicate that PEE partly inhibits the Th17/IL-17 axis in psoriasis-like skin lesions.

### 2.7. A Comprehensive Investigation into the Mechanism of PEE in Inhibiting the Th17/IL-17 Axis Through Integrated Network Pharmacology and Transcriptomic Analysis

**Figure 7:**
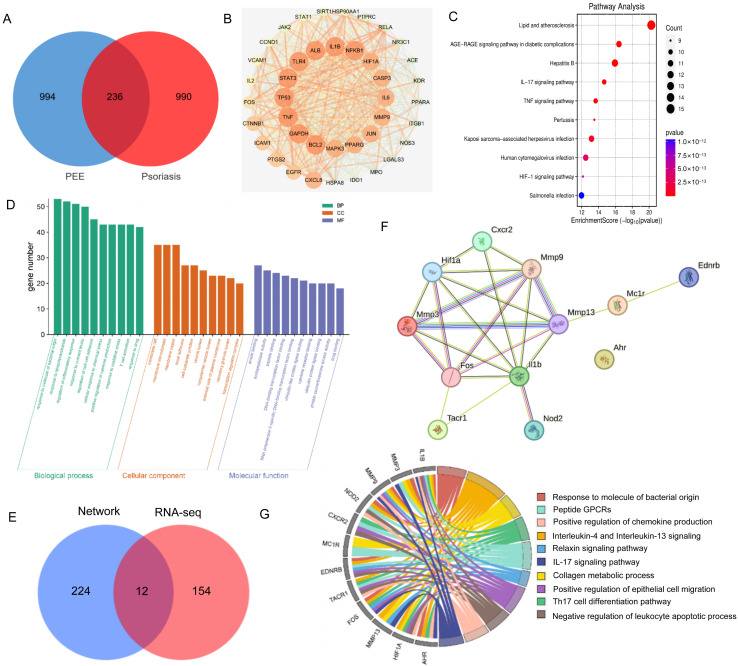
Integrating network pharmacology and transcriptomics to explore the potential mechanism of PEE in blocking the Th17/IL-17 axis. (**A**) Common targets between PEE and psoriasis. (**B**) PPI network layout analysis of core targets. Nodes correspond to target proteins, and each node’s color indicates the degree of interconnection. Edges between nodes symbolize protein–protein interactions within the network. (**C**) KEGG term enrichment analysis. (**D**) GO term enrichment analysis. (**E**) Intersection targets between network pharmacology and transcriptomics. (**F**) Visualization of intersection targets. (**G**) String diagram of intersection target enrichment analysis.

To explore the relationship between the components of PEE and their targets in the treatment of psoriasis, an integrated network pharmacology and transcriptomic analysis was employed. A total of 2872 compounds were identified from PEE by metabolomics analysis. Then, 1370 compounds among these were subjected to target prediction using the Swiss Target Prediction database, with a screening criterion of Probability > 0.5. This analysis yielded 1230 potential targets. Then, we identified 1226 genes associated with psoriasis from the GeneCards, OMIM, and DisGeNET databases. Intersection analysis identified 236 common targets between PEE targets and genes associated with psoriasis. These common targets were considered potential targets for PEE in the treatment of psoriasis (Figure 7A). To identify core targets, we constructed a protein–protein interaction (PPI) network using the String database. Based on the criteria of Closeness > 0.0022, Betweenness > 222.0606, and Degree > 41.6623, 41 core targets were screened (Figure 7B). Meanwhile, GO and KEGG pathway analyses were conducted to further investigate and explain the potential mechanisms underlying their predicted actions. The GO analysis revealed significant enrichment of genes involved in the response to bacterial molecules, response to lipopolysaccharides, regulation of the inflammatory response, response to nutrient levels, and regulation of cell–cell adhesion (Figure 7C). KEGG enrichment results indicated that these targets were primarily enriched in blood lipid metabolism and atherosclerosis, the effects of the AGE-RAGE signaling pathway in diabetic complications, hepatitis B, the IL-17 signaling pathway, and the TNF signaling pathway (Figure 7D). GO and KEGG enrichment analyses indicated that PEE could modulate pathways involved in psoriasis pathogenesis. To identify the key targets of PEE for the treatment of psoriasis, an integrated network pharmacology and transcriptomics analysis was employed (Figure 7E). Through an in-depth analysis of the aforementioned network pharmacology and transcriptomics, the results indicated that CXCR2, HIF1A, MMP9, MMP3, MMP13, FOS, IL1B, TACR1, NOD2, MC1R, EDNRB, and AhR were potential key targets of PEE for treating psoriasis (Figure 7F). Further analysis revealed pathways regulated by these 12 key genes, including critical pathways associated with psoriasis, such as the positive regulation of chemokine production, IL-4 and IL-13 signaling, the IL-17 signaling pathway, and the Th17 cell differentiation pathway (Figure 7G). In summary, the integrated analysis of network pharmacology and transcriptomics identified 12 key targets through which PEE may block the Th17/IL-17 axis to treat psoriasis.

### 2.8. AhR Was a Key Target of PEE for Blocking the Th17/IL-17 Axis

**Figure 8:**
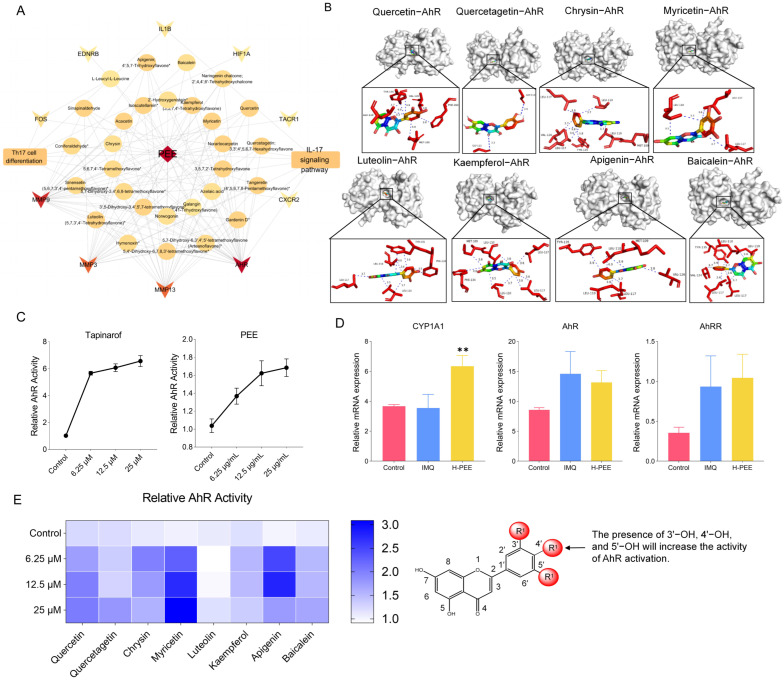
PEE and its constituent compounds activated AhR. (**A**) Interaction map of key targets and their associated components. Network pharmacology analysis identifies core targets through which the primary flavonoid components of PEE improve psoriasis-like skin lesions in mice. *: Compounds marked with asterisk are characteristic constituents from PPL. (**B**) Molecular docking results of quercetin, quercetin marigold, poplarin, myricetin, luteolin, kaempferol, apigenin, and baicalin with AhR. (**C**) The AhR activating activities of PEE and the positive drug tapinarof. (**D**) The effect of PEE on the AhR downstream gene CYP1A1 in psoriatic skin lesions. n = 3 samples per group; data are expressed as mean ± SD. ** *p* < 0.01, compared to IMQ. Statistical significance was calculated using one-way ANOVA. (**E**) The effect of quercetin, quercetin-2,3-diol, poplarin, myricetin, luteolin, apigenin, kaempferol, and baicalin on AhR activity.

Furthermore, we explored the targets of PEE in blocking the Th17/IL-17 axis. Based on the integrated network pharmacology and transcriptomics analysis, the targets regulating the IL-17 signaling pathway include MMP9, MMP3, MMP13, FOS, IL1B, and AhR. In contrast, the targets for regulating Th17 cell differentiation include CXCR2, HIF1A, FOS, IL1B, TACR1, NOD2, and AhR (Figure 8A). We focus on AhR, a novel target for the treatment of psoriasis. Using network pharmacology, we found that quercetin, quercetagetin, chrysin, myricetin, luteolin, kaempferol, apigenin, and baicalein can bind to AhR. To evaluate the ligand–receptor interactions between the eight components in PEE and AhR, we performed molecular docking simulations. The binding sites of these compounds with AhR were visualized using the PLIP platform and PyMOL v.3.8 software. The binding modes for quercetin, quercetagetin, chrysin, myricetin, luteolin, kaempferol, apigenin, and baicalein with AhR are shown in Figure 8B. Meanwhile, to efficiently assess the AhR-activating capacity of individual compounds, we used HaCaT cell lines stably expressing the AhR-mediated reporter gene, previously established by our research group. We found that PEE activated AhR in a concentration-dependent manner (Figure 8C). Tapinarof, the positive control, showed approximately 6-fold AhR activation compared to the blank group (Figure 8C). Additionally, PEE induced the expression of the AhR-targeted gene CYP1A1 in mouse psoriasis-like skin lesions (Figure 8D), indicating that PEE activated AhR in vivo. Furthermore, we examined the AhR-activating activity of the components predicted by network pharmacology. Among the 8 compounds in PEE tested at 12.5 μM, quercetin, myricetin, and apigenin showed significant AhR activation exceeding 2-fold. In comparison, the other five compounds (quercetagetin, chrysin, luteolin, kaempferol, and baicalein) displayed weaker activation effects (Figure 8E). Interestingly, structure-activity relationship (SAR) analysis of these 8 compounds revealed that the spatial topology of the R1 substituents consistently enhanced AhR activation (Figure 8E). In conclusion, PEE activated AhR, which may be a crucial mechanism underlying its blockade of the Th17/IL-17 axis.

### 2.9. Neuronal-Related Pathways Represent Novel Potential Mechanisms for PEE in Alleviating Psoriatic Lesions

**Figure 9:**
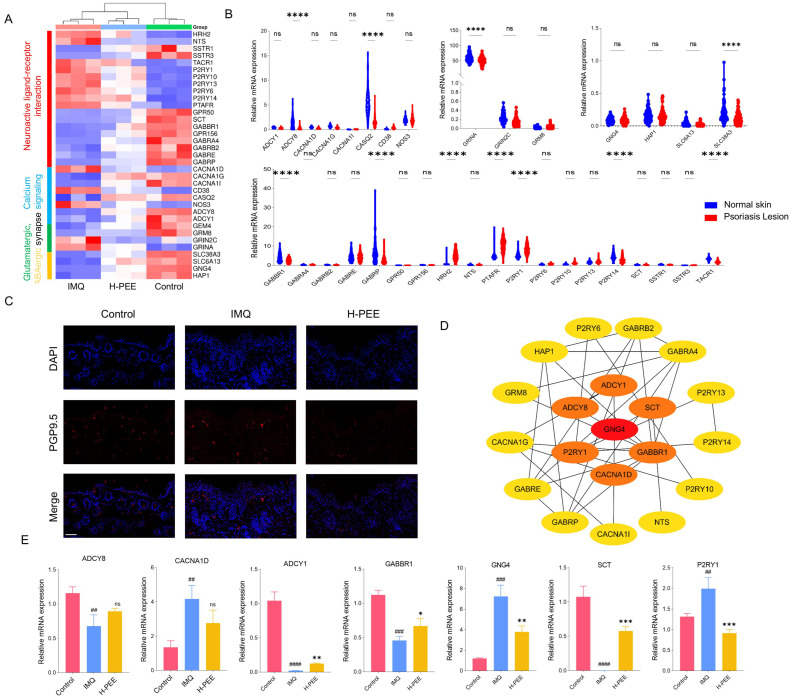
H-PEE regulated neuronal-related pathways in psoriasis-like skin lesions. (**A**) Heatmap of differentially expressed genes (DEGs) in the neuronal-related pathways from transcriptomic results. (**B**) The expression of DEGs in (**A**) in the skin lesions of psoriasis patients. (**C**) The distribution of neurons in psoriasis-like skin lesions. Neurons were visualized using Nissl staining and immunofluorescence staining of PGP9.5. In Nissl staining, neurons are indicated by red arrows, and the scale bar represents 100 μm. In the immunofluorescence staining of PGP9.5, neurons are stained red, and the scale bar represents 20 μm. (**D**) The protein–protein interaction network based on genes differentially expressed in psoriasis patients was analyzed. (**E**) RT-qPCR was used to detect the mRNA expression levels of ADCY8, CACNA1D, ADCY1, GABBR1, GNG4, SCT, and P2RY1 in skin tissue. n = 5 samples per group; data are presented as mean ± SD. ns: no significant difference, ## *p* < 0.01, ### *p* < 0.001, #### *p* < 0.0001, compared to control; * *p* < 0.05, ** *p* < 0.01, *** *p* < 0.001, **** *p* < 0.0001, compared to IMQ. Statistical significance was calculated using two-way ANOVA.

As mentioned above, transcriptome analysis revealed significant enrichment in the neuroactive ligand–receptor interaction pathway. Additionally, several other neuronal-related pathways, such as the “glutamatergic synapse,” “calcium signaling pathway,” and “GABAergic synapse,” were also enriched. This finding suggests that neuronal-related pathways may play a previously underappreciated role in the pathogenesis and treatment of psoriasis. To systematically visualize the expression patterns of neuronal-related genes, the differentially expressed genes (DEGs) in these pathways were displayed as heatmaps (Figure 9A). The results revealed significant differences in the expression of neuronal genes between the IMQ and control groups (Figure 9A). Notably, the H-PEE group successfully reverted most abnormally expressed neuronal-related genes to normal levels (Figure 9A). Furthermore, to ensure the clinical relevance of our findings, the transcriptomic dataset for psoriatic patients (GSE54456) was downloaded from the GEO database to examine trends in neuronal-related DEGs identified in the mouse model. Encouragingly, the expression trends of the vast majority of neuronal-related DEGs were consistent between psoriatic lesions in mice and human psoriasis (Figure 9B), suggesting that the abnormal neuronal activity is a key pathological mechanism in psoriasis. The results provide strong evidence that neuronal pathways play a previously underappreciated role in PEE-mediated psoriasis treatment.

Based on these transcriptomic findings, we performed PGP9.5 immunohistochemistry (IHC) staining to visualize the neuronal distribution in psoriatic lesions. Compared to the control group, the IMQ group exhibited significantly increased densities of PGP9.5-positive nerve fibers in the dermis. Following H-PEE treatment, the densities of both neural markers were significantly downregulated, returning to near-normal levels (Figure 9C). This finding directly confirms that H-PEE can partly reverse the abnormal neuronal activity associated with psoriatic lesions.

Furthermore, to identify the most central regulatory nodes within the complex gene network, the neuronal-associated differentially expressed genes (DEGs) were input into the STRING database to construct a protein–protein interaction (PPI) network (Figure 9D). By analyzing network topological parameters (connectivity), we identified six hub genes: NTS, P2RY1, GNG4, GABRP, GABBR1, and ADCY8 (Figure 9D). The expression levels of these six hub genes in psoriatic lesions were then detected via RT-qPCR. These results correlated highly with the RNA-seq data (Figure 9E). Specifically, compared with the control group, the mRNA expression of NTS, P2RY1, and GNG4 was significantly upregulated in the IMQ group, whereas the expression of GABRP, GABBR1, and ADCY8 was downregulated. H-PEE treatment significantly reversed the abnormal expression of these key genes, restoring them toward normal levels. These results further confirmed that neuronal-related pathways represent novel potential mechanisms for PEE in alleviating psoriatic lesions.

## 3. Discussion

PEE alleviated primary psoriasis-like skin lesions and reduced the occurrence of recurrent lesions in mice, which was characterized by inhibition of abnormal keratinocyte proliferation and remodeling of dermal structure. Mechanistically, PEE modulated the balance between Th17/Treg cells and partially inhibited IL-17 signaling, thereby blocking the Th17/IL-17 axis, which is the core molecular pathological mechanism underlying the development and progression of psoriasis. AhR was identified as a potential target of PEE for blocking the Th17/IL-17 axis mediated by several flavonoid components. Additionally, PEE disrupted neuronal-related pathways, representing a novel potential mechanism for alleviating psoriasis-like skin lesions. Most importantly, in the process of improving psoriasis with PEE, PEE simultaneously activated AhR and inhibited the Th17/IL-17 axis and neuronal-related pathways, which may represent a novel mechanism of PEE in the treatment of psoriasis. For the first time, our study comprehensively investigated the potential of pomegranate peel in treating psoriasis.

Recent research suggests that psoriasis is not merely a localized skin lesion of chronic inflammation; its progression can trigger systemic inflammatory cascades, significantly increasing the risk of systemic comorbidities [19,20]. Notably, the pathophysiological mechanisms of this disease exhibit intersections in molecular pathways (e.g., JAK-STAT signaling pathway and Th17 cell differentiation), with various autoimmune diseases (e.g., rheumatoid arthritis, inflammatory bowel disease) and autoinflammatory diseases, suggesting that it may serve as an important phenotypic window for systemic immune dysregulation [21,22]. As recent studies indicate, even with the clinical use of biologics to alleviate psoriasis, where 60% of patients achieve near-complete or complete skin clearance, a high recurrence rate persists—particularly with the reappearance of lesions following treatment discontinuation [23]. The median time to relapse after discontinuing traditional immunosuppressants (such as methotrexate or cyclosporine) is approximately 4 weeks, whereas the median relapse time for biologics is significantly prolonged to 12–34 weeks [24]. Currently, although multiple treatment options exist for primary and recurrent psoriasis, critical challenges, including drug-related safety risks (e.g., cutaneous/systemic adverse reactions), acquired drug resistance, long-term treatment cost burdens, and individual efficacy variability, remain urgent issues to address [25]. Against this backdrop, developing novel therapeutic agents for psoriasis has become an urgent priority.

Pomegranate peel is not only a food waste product but also a renowned traditional Chinese medicine commonly used in folk practices to treat acne, psoriasis, and other conditions. In a study exploring the overall medicinal characteristics and formulation principles of external washing formulas from the Guangdong Provincial Hospital of Traditional Chinese Medicine for treating psoriasis vulgaris, it was found that the most frequently used Chinese medicinal herb was pomegranate peel [26]. However, research on the topical application of pomegranate peel for the treatment of psoriasis remains limited. In this study, the therapeutic effects of pomegranate peel on both primary and recurrent psoriasis, as well as its underlying mechanisms, were thoroughly investigated, which is of significant importance for developing pomegranate peel into a popular formulation for the treatment of psoriasis. Pomegranate extract (PEE) modulates the balance between Th17/Treg cells and inhibits IL-17 signaling, thereby blocking the Th17/IL-17 axis. Pomegranate peel inhibits the PI3K/Akt, MAPK, and NF-κB pathways to exert anti-inflammatory effects, which may be one of the mechanisms by which pomegranate peel treats psoriasis. Its specific therapeutic role in psoriasis via Th17/Treg modulation and IL-17 inhibition had not been elucidated prior to this study [17,27,28].

The effect of pomegranate peel on the Th17/IL-17 axis has been studied in tissues other than the skin. In a model of human peripheral blood mononuclear cell culture, pomegranate peel extract inhibits the differentiation of T cells into Th17 cells, thereby promoting their differentiation into Treg cells [29]. In a model of DSS-induced colitis, pomegranate peel extract reduces the level of IL-17 in intestinal inflammatory tissue [30]. These findings indicate that pomegranate peel extract can block the Th17/IL-17 axis, consistent with our findings. The Th17/IL-17 axis is the core molecular pathological mechanism underlying the development and progression of psoriasis, and biologics targeting this axis have achieved remarkable success in psoriasis treatment [31]. Therefore, based on the inhibition of Th17/IL-17 by pomegranate peel extract, it is supported that this extract has great potential to be developed into a therapeutic agent for psoriasis.

We identified AhR as a potential target of PEE for blocking the Th17/IL-17 axis. There is limited research on the key targets of PEE in inhibiting the Th17/IL-17 axis. The most extensively studied component is punicalagin, which is the key component of pomegranate peel [32]. Punicalagin decreases the production of cytokines by Th17 cells by inhibiting the NF-κB signaling pathway [33]. Additionally, punicalagin enhances AhR transcriptional expression to promote the anti-inflammatory response in macrophages [34]. In this study, molecular docking and luciferase reporter gene assays confirmed that eight compounds, including quercetin, quercetagetin, chrysin, myricetin, luteolin, kaempferol, apigenin, and baicalein, can activate AhR.

Further literature reviews indicate that the aforementioned AhR-activating compounds demonstrate significant anti-inflammatory and therapeutic potential in psoriasis models. Specifically, quercetin suppresses excessive keratinocyte proliferation and alleviates psoriatic pathology by inhibiting the Notch and PI3K/AKT signaling pathways while inducing Glut1-mediated keratinocyte apoptosis [35]. Quercetagetin has been reported to inhibit inflammatory responses and oxidative stress [36]. Chrysin improves psoriasiform skin lesions in mice, while its structural derivatives further alleviate skin inflammation by suppressing NF-κB and STAT3 signaling pathways [37]. Myricetin significantly alleviates skin inflammation in mice by regulating macrophage polarization: it inhibits the STAT1 and NF-κB pathways to block M1 polarization, while activating the STAT6 pathway to promote M2 polarization [38]. Local administration of luteolin via a nanostructured lipid carrier gel effectively improves imiquimod-induced psoriasiform symptoms in mice [39]. Although kaempferol possesses anti-inflammatory, antioxidant, and antiproliferative properties, its poor water solubility and low bioavailability limit its application. The development of a kaempferol hydrogel formulation effectively overcomes this limitation and demonstrates promising therapeutic effects in psoriasis animal models [40]. Apigenin improves psoriasis-like phenotypes by inhibiting the abnormal proliferation of keratinocytes through suppression of the CDK2/E2F2 pathway and by exerting anti-inflammatory effects [41]. Furthermore, baicalin not only possesses anti-inflammatory and antioxidant capabilities but also inhibits Th17 cell differentiation and IL-17A production in vitro, suggesting that it may exert therapeutic effects by regulating immune cell function [42]. Notably, these observed therapeutic effects align closely with the core immunomodulatory function of AhR activation. AhR promotes the differentiation of Treg cells and inhibits the differentiation of Th17 cells through direct or indirect pathways [43]. Therefore, this study confirms that AhR may be a new target for the treatment of psoriasis with pomegranate peel, which is crucial for elucidating the scientific basis of pomegranate peel in treating psoriasis. However, further in-depth and systematic research is still required to identify the specific components in pomegranate peel that can activate AhR, the modes of action of each component on AhR, and the direct role of AhR in psoriasis also needs to be further verified.

The disruption of neuronal-related pathways has been identified as a novel potential mechanism for PEE in alleviating psoriasis-like skin lesions. Recent research indicates that neurogenic inflammation is a newly identified pathogenic mechanism in psoriasis. In psoriatic lesions, sensory neurons increase in number around keratinocytes and immune cells [44]. The sensory ASIC3 channel exacerbates psoriatic inflammation by inducing sensory neurons to release CGRP. This neuroactive ligand acts on dendritic cells to stimulate IL-23 production and also promotes the proliferation of skin keratinocytes by binding to the CGRP receptor [45]. In psoriasis-like skin lesions, neuroceptors (such as TRPV1) mediate neurite outgrowth induced by Tnc+ fibroblasts, thereby promoting close contact and interaction between peripheral nerves and T cells, leading to excessive proliferation of keratinocytes and infiltration of immune cells [46]. These studies collectively demonstrate that sensory neurons actively drive the cascade amplification of the IL-23/IL-17 inflammatory axis through neuropeptide release (e.g., CGRP) and structural remodeling (e.g., TRPV1-mediated neurite outgrowth). Neurogenic signaling not only triggers immune cell activation but also serves as a core hub in psoriasis, directly regulating keratinocyte proliferation and immune infiltration [47,48].

These studies collectively support the novel therapeutic approach of targeting neuronal pathways as a potential means of alleviating psoriasiform skin lesions in PEE. Our study found that in IMQ-induced psoriasis-like skin lesions, the number of neurons increased, and the expression of certain neuroactive ligands (such as NTS) and neuroactive receptors (such as P2RYs) was upregulated. In contrast, the expression of other neuroactive ligands (such as SCT) and neuroactive receptors (such as GABRs) was downregulated. PEE partially reversed neuronal growth and changes in neuroactive ligands and receptors in psoriasis-like skin lesions. In patients with psoriasis, serum NT levels were elevated, leading to mast cell release of VEGF, an important inflammatory factor in the progressive stage of psoriasis [49]. P2RYs are expressed in keratinocytes, fibroblasts, and primary sensory nerves of the skin, and are involved in the pathological process of psoriasis-related skin pruritus [50]. The GABAR ligand, GABA, is decreased in the serum of psoriasis patients and regulates the activation of peripheral blood T cells, thereby suppressing the immune response [51]. In summary, the trends in neuroactive ligands and receptors observed in this psoriasis mouse model are consistent with those reported in psoriasis patients in the literature. However, most current research focuses on the expression of neuroactive ligands and receptors in psoriasis.

However, this study has several limitations. First, the batch-to-batch consistency of pomegranate peel, the selection of marker compounds, and the quantitative characterization of PEE remain to be further clarified, which is crucial for precise quality control and stable therapeutic efficacy. Second, the animal model used in this study only partially reflects the pathological features of human psoriasis and thus has limitations in simulating the complex pathogenesis and comorbidities of clinical psoriasis. Then, a few studies have examined targeting neuroactive ligands and receptors for psoriasis treatment. This study found that PEE can reverse the expression of neuroactive ligands and receptors in psoriatic lesions, providing an opportunity to develop psoriasis treatments that target neuroactive ligand–receptor interactions. Further research on PEE should focus on its active components and the mechanisms by which it regulates neuroactive ligand–receptor interactions. Finally, there are also many limitations in the use of experimental techniques. IL-23R alone may not fully define Th17 populations; RORγt is also a common choice. For animal models of psoriasis, IMQ-induced models do not reflect the real pathological conditions of psoriasis, and a variety of transgenic models are recommended.

## 4. Materials and Methods

### 4.1. Preparation of PEE

Initially, pomegranate peel powder was macerated in 75% ethanol (*v*/*v*) for one week, followed by ultrasonic extraction for 2 h and filtration. The residue was subjected to a second ultrasonic extraction with 75% ethanol for 2 h and filtered again. The filtrates were combined and concentrated into an extract using rotary evaporation. The extract was transferred to an evaporating dish to remove residual solvent and then freeze-dried to obtain the pomegranate extract (PEE).

### 4.2. Metabolomics Analysis of PEE

Non-targeted metabolomics analysis was conducted by Wuhan Maiwei Metabolomics Technology Co., Ltd., Wuhan China. The specific experimental workflow, including sample preparation, chromatographic-mass spectrometry analysis conditions, and data preprocessing methods, is described as reported in previous studies [52]. Compound quantification was performed in MRM mode on a triple quadrupole mass spectrometer to obtain spectral data. Subsequently, the peak areas of all chromatographic peaks were integrated to derive the relative content of each compound for further analysis.

### 4.3. Primary and Recurrent Mouse Models of Psoriasis

Healthy male Balb/c mice (7 weeks old) used in this study were provided by Beijing Vital River Laboratory Animal Technology Co., Ltd., Beijing China. Animal experiments strictly adhered to bioethical standards and were approved by the Research Ethics Committee of Shanxi University (Approval No.: SXULL2022019). Housing conditions were maintained as follows: room temperature was controlled at 22 ± 2 °C, relative humidity was maintained between 45% and 55%, and a 12 h light–dark cycle with natural light patterns was implemented. Experimental animals had free access to standard laboratory chow and drinking water. The tested drugs were dissolved in a solvent mixture of 70% ethanol (*v*/*v*%), propylene glycol, and ddH_2_O at a volume ratio of 7:1:2 (*v*/*v*/*v*).

Firstly, the therapeutic effect of PEE on primary psoriasis was evaluated. The dorsal hair of mice was shaved, and depilatory cream was used to remove residual hair. The mice were randomly divided into six groups (n = 5 per group) according to body weight: control group, IMQ group, Tapinarof group, H-PEE group, M-PEE group, and L-PEE group. The control group was treated with a blank matrix on the dorsal skin, while the other groups were topically applied 62.5 mg of imiquimod (IMQ) cream on the back each morning to establish psoriasis-like lesions. Eight hours later, the control and IMQ groups were treated with the drug vehicle, and the Tapinarof, H-PEE, M-PEE, and L-PEE groups were administered the corresponding therapeutic agents. The administration volume was 60 μL, and the concentrations of H-PEE, M-PEE, and L-PEE were 10%, 5%, and 1% (m/m%), respectively. All groups were treated continuously for 7 days using the protocol described above.

Next, to evaluate the effect of drugs on psoriasis recurrence, a mouse model of recurrent psoriasis was established. After hair removal, mice were randomly assigned to groups based on body weight: control, control (low), IMQ, Tapinarof, and H-PEE. Psoriasis-like lesions were induced with IMQ and treated with the designated agents as described above; the control (low) group received the same treatment as the control group. No additional interventions were performed from day 8 to day 20, allowing spontaneous recovery of psoriasis-like lesions. Afterward, the regrown dorsal hair was removed, and all mice were kept in the same groupings as in the initial modeling phase for long-term observation. Except for the control group, all other groups were topically treated with 22.5 mg IMQ cream on the dorsal skin for 4 consecutive days to induce recurrence of psoriasis-like lesions.

The Psoriasis Area and Severity Index (PASI) was used to conduct daily assessments and record the severity of psoriasis-like skin lesions in mice, specifically erythema, scaling, and skin thickness. Detailed scoring rules are provided in Table S1 [53].

### 4.4. H&E Staining

Skin tissues harvested from mice were fixed in 4% paraformaldehyde for at least 24 h, followed by graded ethanol dehydration and paraffin embedding. Subsequently, 5 μm-thick sections were prepared using a microtome. After dewaxing in xylene and rehydration through a graded ethanol series, the sections were stained with hematoxylin and eosin (H&E), dehydrated in anhydrous ethanol, cleared in xylene, and mounted with a resinous medium. Skin tissue sections were examined by light microscopy for histopathological analysis. ImageJ was used to perform quantitative analyses of epidermal thickness, the number of epidermal ridges, and the number of infiltrating inflammatory cells.

### 4.5. Immunohistochemistry Staining

Immunohistochemical (IHC) analysis was performed on skin tissue sections to assess Ki-67 expression. Deparaffinized sections underwent antigen retrieval. The tissue sections were incubated with 3% hydrogen peroxide to block endogenous peroxidase activity. Subsequently, the sections were incubated overnight at 4 °C with the Ki67 primary antibody (ab16667, diluted 1:200, Abcam, Cambridge, MA, USA). After washing, the sections were incubated with HRP-conjugated secondary antibodies for 60 min at 37 °C. Following this, the sections were treated with a sufficient volume of freshly prepared 3,3′-diaminobenzidine chromogen solution. After the final wash, the sections were counterstained with hematoxylin.

### 4.6. Immunofluorescence Staining

For fluorescent single-label staining, deparaffinized skin tissue sections underwent antigen retrieval, followed by PBS washes (pH 7.4). The skin tissue sections were blocked with species-appropriate serum or BSA, incubated overnight at 4 °C with the primary antibody (anti-PGP9.5, GB11277, 1:200, Servicebio, Wuhan China), washed, and treated with a fluorophore-conjugated secondary antibody at room temperature. Nuclei were counterstained with DAPI. Autofluorescence quenching was performed as needed. After covering the sections with an anti-fading mounting medium, fluorescence images were acquired using a preset filter set.

For fluorescent double staining, the tyramide signal amplification method was applied. After overnight incubation at 4 °C in a humidified chamber with the primary antibodies (anti-Foxp3, GB15064, 1:2000, Servicebio; anti-IL-23R, GB11660, 1:5000, Servicebio), the sections were washed with PBS the following day and then incubated at room temperature with the corresponding HRP-labeled secondary antibody. The sections were subsequently incubated with tyramide signal amplification reagent in the dark. Following the first tyramide signal amplification, the sections were labeled with the second secondary antibody (IL-23R antibody). Finally, the sections were stained for nuclei, mounted, and photographed. Images were analyzed using ImageJ (Version 1.46r) to quantify Foxp3- and IL-23R-positive cells.

### 4.7. Transcriptomics

Skin tissue samples from the dorsal region of mice were immediately cryopreserved upon collection and shipped to Shanghai Paisenuo Biotechnology Co., Ltd., Shanghai, China for transcriptomic sequencing following standard procedures. Total RNA from the skin tissue was extracted using the TRIzol method and subjected to quality control. mRNA was enriched using Oligo(dT) magnetic beads, followed by fragmentation and double-stranded cDNA synthesis. The purified cDNA underwent end modification and adapter ligation to construct the library. Following fragment selection, amplification, and quality control, PE150 sequencing was performed on the Illumina platform. Differential gene expression analysis was conducted using DESeq, with the following criteria for screening differentially expressed genes (DEGs): |log2FoldChange| > 1 and *p*-value < 0.05.

### 4.8. Bioinformatics Analysis of Transcriptome Results

For Gene Ontology (GO) and Kyoto Encyclopedia of Genes and Genomes (KEGG) enrichment, DEGs were analyzed using the GenesCloud software (Version 1, Shanghai Paisenuo Biotechnology Co., Ltd., Shanghai, China) (https://www.genescloud.cn/home, accessed on 02 Dec 2025). For Ingenuity Pathway Analysis (IPA), DEGs were analyzed with IPA software (v4.13.1, QIAGEN, Redwood City, CA, USA) across four modules: canonical pathways, disease and biological function, upstream regulators, and toxicity functions. For immune cell infiltration analysis, DEGs were analyzed with the ImmuCellAI-mouse tool (https://guolab.wchscu.cn/ImmuCellAI-mouse/#!/, accessed on 20 December 2025).

### 4.9. Real-Time Quantitative PCR Analysis (qPCR)

Total RNA was extracted from mouse skin tissue samples and reverse transcribed to cDNA using the SweScript All-in-One RT SuperMix for qPCR Kit (Servicebio, Wuhan, China; catalog no. G3337-100). Quantitative PCR (qPCR) was subsequently performed using the 2× Universal Blue SYBR Green qPCR Master Mix (Servicebio, Wuhan, China; catalog no. G3326-15), with β-actin (ACTB) mRNA serving as the internal reference gene for normalization of target gene expression levels. The primer sequences are provided in Table S2.

### 4.10. Network Pharmacology

Metabolomic analysis was used to identify the chemical composition of PEE. Precursor ion mass tolerance was set at ±5 ppm. Compounds were selected based on Level 2 identification confidence, which required matching scores greater than 0.7 for both secondary mass spectra and retention time against reference databases, as well as the presence of valid CAS registry numbers. Potential biological targets of the filtered compounds were subsequently predicted using the SwissADME (http://www.swissadme.ch/, accessed on 10 September 2024) platform. Target genes associated with psoriasis were obtained from the GeneCards (https://www.genecards.org/), Online Mendelian Inheritance in Man (OMIM, https://www.omim.org/), and DisGeNET (https://www.disgenet.org/) databases, and the targets were screened based on the median score. The targets of PEE for treating psoriasis were identified using Venn analysis to determine the intersection between psoriasis-related genes and the PEE targets.

The targets of PEE were imported into the STRING database (https://cn.string-db.org/) to construct the protein–protein interaction (PPI) network, and Cytoscape 3.9.1 software was used to visualize the network topology. Core targets were screened based on closeness, betweenness, and degree values. After optimizing the node layout (color, shape, and connections), the image was exported. Finally, the PPI network diagram, screened target diagram, and key target matrix diagram were integrated for comprehensive analysis.

### 4.11. Integrated Analysis of Transcriptomics and Network Pharmacology

The core targets were defined as the intersection of differentially expressed genes identified from transcriptomic analysis and potential targets predicted by network pharmacology. Subsequently, a PPI network was constructed using these core targets in the STRING database (https://cn.string-db.org/). Functional enrichment analysis (including GO terms and KEGG pathways) was then performed for the core targets using the Metascape platform (Version 3.5, https://metascape.org/gp/index.html) to elucidate the principal biological processes and signaling pathways modulated by these targets.

### 4.12. Molecular Docking

In PyMOL 3.8 software, we performed molecular modifications on protein structures downloaded from the RCSB PDB database (https://www.rcsb.org/), removing water molecules and non-essential components. The structures were hydrogenated and charge-optimized using AutoDockTools (Version 1.5.7) to generate PDBQT files. Ligands in SDF format were downloaded from the PubChem database (https://pubchem.ncbi.nlm.nih.gov), optimized with Chem3D, and converted to mol2 format. Rotatable bonds were identified using AutoDockTools, and a PDBQT file was generated. The binding site was defined to facilitate molecular docking. The PLIP platform and PyMOL were used to analyze the interaction site, and complexes with binding energies ≤ −5 kJ/mol were screened.

### 4.13. AhR Activity Assay

The luciferase reporter gene assay was used to assess AhR activity. The AhR-mediated luciferase reporter gene was transfected into HaCat cells to generate HaCat (AhR-Luc) cells. HaCat (AhR-Luc) cells were cultured in DMEM medium supplemented with 10% FBS and 1% penicillin-streptomycin. The cultivation was carried out at 37 °C in a 5% CO_2_ atmosphere. HaCat (AhR-Luc) cells were seeded in 96-well plates and treated with increasing concentrations of compounds (luteolin, apigenin, baicalein, quercetin, quercetagetin, chrysin, kaempferol, or myricetin). After 24 h of incubation, cell luciferase activity was detected using the Luciferase Reporter Assay System (Beyotime, Wuhan, China) according to the manufacturer’s instructions.

### 4.14. ELISA Assay

Total proteins were extracted and analyzed to detect cytokine levels, including IL-17A, IL-6, TNF-α, and IL-23. The mouse IL-6 and TNF-α ELISA assay kits were obtained from Wuhan Abclonal Technology Co., Ltd., Wuhan, China while the mouse IL-23 and IL-17A ELISA assay kits were sourced from Multisciences Biotech Co., Ltd., Hangzhou, China.

### 4.15. Masson’s Trichrome Stain

Skin tissues were collected from mice and immediately fixed in pre-cooled 4% paraformaldehyde for 24 h at room temperature. The fixed tissues were then subjected to routine processing: gradient ethanol dehydration, xylene clearing, and paraffin embedding. Paraffin blocks were sectioned at 5 μm thickness using a rotary microtome. Sections were baked at 60 °C for 1 h, deparaffinized in xylene, and rehydrated through a descending ethanol series. Masson trichrome staining was performed as follows: sections were counterstained with hematoxylin, stained with ponceau-acid fuchsin solution, treated with phosphomolybdic acid, differentiated, dehydrated, and mounted with resin medium. Histopathological observation was conducted under an optical microscope. Collagen fiber area percentage was quantified using ImageJ software, with uniform threshold parameters applied across all samples.

### 4.16. Statistical Analysis

Data were expressed as mean ± standard deviation (SD). Statistical differences were analyzed using either a one-way ANOVA or a two-way ANOVA in GraphPad Prism (Version 9.1.0), with *p* < 0.05 considered statistically significant.

## 5. Conclusions

In conclusion, our study demonstrated that PEE topically alleviated primary psoriatic skin lesions and reduced lesion recurrence by suppressing the expression of key cytokines involved in psoriasis pathogenesis. Mechanistically, PEE activated AhR, which may be a potential target for PEE to inhibit the Th17/IL-17 axis. Additionally, transcriptomic analysis revealed neuronal-related pathways as novel potential mechanisms for the alleviation of psoriasis-like skin lesions by PEE. This research established PEE as a novel therapeutic agent for psoriasis with a unique mechanism of action. Furthermore, it indicated that pomegranate peel holds significant potential for translation and application, playing an important role in the sustainable reuse of waste materials in the pomegranate industry.

## Data Availability

The original contributions presented in this study are included in the article/supplementary material. Further inquiries can be directed to the corresponding author.

## Supplementary Materials

The following supporting information can be downloaded at https://www.mdpi.com/article/10.3390/ijms27125171/s1.

## Abbreviations

The following abbreviations are used in this manuscript:

PEE	pomegranate peel ethanol extract
IMQ	imiquimod
PASI	psoriasis area and severity index
AhR	aryl hydrocarbon receptor
GO	Gene Ontology
KEGG	Kyoto Encyclopedia of Genes and Genomes
PPI	protein–protein interaction
H&E staining	hematoxylin and eosin staining
IHC	Immunohistochemistry
DEGs	differentially expressed genes
SD	standard deviation
TIC	total ion chromatogram
IPA	Ingenuity Pathway Analysis
qPCR	quantitative real-time PCR analysis
