# Supplementary material for: Pomegranate Peel Extract Alleviates Psoriasis-like Skin Lesions in Mice Through the Suppression of AhR-Activating Th17/IL-17 Axis and Neuronal-Related Pathways
Source: Int J Mol Sci. 2026 Jun 7;27(12):5171. doi: 10.3390/ijms27125171 (PMC13299329; doi:10.3390/ijms27125171)
Supplement: Supplementary file 1 [file ijms-27-05171-s001.zip › Supplemental Figures.pdf]

Supplemental materials

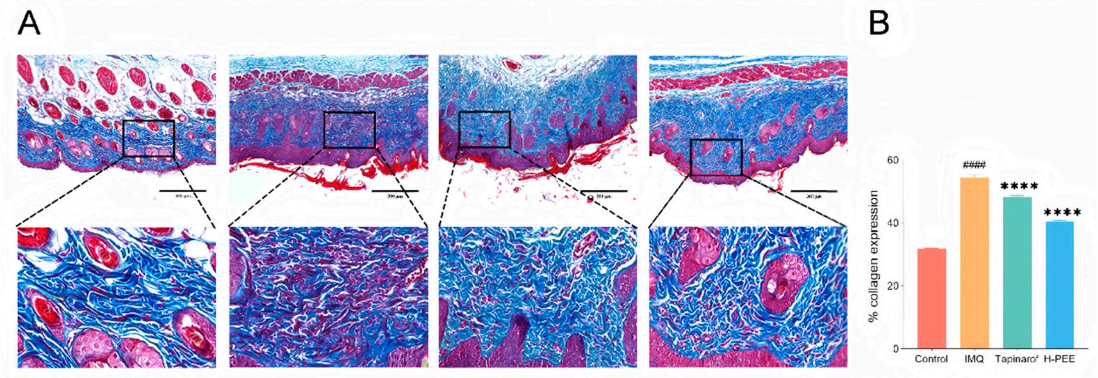

Figure S1. Masson staining results. (A,B) Skin samples were subjected to Masson staining to assess the percentage of collagen expression, and statistical analysis was performed on collagen expression in different groups.
